# Sulphur

**Published:** 1887-10

**Authors:** J. T. Kent


### SULPHUR.

A Lecture by Professor J. T. Kent, A. M., M. D.

(Continued from September number, page 329.)

The condition of the eyes produced by Sulphur is similar to that produced by many other remedies. Inflammation of the lids, yellow or yellowish white, muco-purulent discharge, crusty formation on the margins, obscuration of sight—like a gauze before the eyes—halo round the gas; there is nothing individual about these. The grand characteristic is the acrid discharge and the *burning upon application of water.* All are curable by Sulphur when the red string is applied. Ulceration of the margins of the lid (Graph., Apis, Ars., Sulph.). All the inflammatory symptoms of purulent ophthalmia of the cornea and the lids will be cured by Sulphur when indicated.

The same acrid, burning, offensive discharge may be found in the ear, with excessive burning after syringing them; discharge from the ears after an acute disease, as scarlet fever, with the burning from application of water. You commonly find burning of the soles of the feet, which are puffed and red at night; cannot endure covering; hot head. Many times we find the symptoms nearly all suppressed, and we have a one-sided case. In children we look for the bright red lips and the redness of the mucous membrane wherever seen. It was Sulphur that had been needed in the beginning—it is Sulphur now. Should the child not be properly treated as it continues to grow, you will find *burning in the top of the head, burning* in the *soles*, the *all-gone hungry* feeling at eleven A. M., or it may be a loose stool in the morning *driving her out of bed*. Men who suppress and maltreat these diseases know no better way.

We have the same grand characteristic in acute diseases of the nose—burning *agg.* by water, with catarrhal discharges of bloody mucus. Sulphur is a strong remedy for this symptom of bloody pus or mucus. It is quite safe to prescribe upon the one symptom when you find such a condition in a general epidemic among horses. It may be checked as quickly in a horse as in a man. In discharge of acrid, burning water from the nose compare Ars., Merc., Al. cepa. Allium cepa has an acrid discharge from the nose and a bland discharge from the eyes. It has a cough that is like the tearing of hooks in the larynx. Euphrasia has an acrid discharge from the eyes and a bland discharge from nose, outer surface of the nose becomes filled with black pores. You will find Graphites an important remedy.

All over this country we have hay-fever. During its season people flee to the White Mountains, to Northern Michigan, across the ocean, everywhere, seeking relief. For four years I have cured every case presented to me; you will do the same thing easily and nicely if you follow the *law of cure*.

On my trip East this year a case presented itself that had been in existence eleven years. The man was at Panama eleven years ago, and the hay-fever had followed an attack of Panama-fever. Coming on each year about the 20th of August he had an attack of hay-fever. He had consulted all sorts of pathists as well as some of the best homœopaths in this country. Recognizing the fact that it had followed Panama-fever, knowing the excellence of the prescribers that had gone before, believing without doubt he had taken Carb. veg. (the king remedy after a badly treated fever), I gave him the sister remedy, Psorinum, the symptoms perfectly agreeing. The disease left him in twenty-four hours and did not return.

Each remedy has its own symptoms. No other remedy will cure an Al. cepa case except Al. cepa, and you cannot make up for your ignorance by increasing the size of the dose. If you would find your remedy you must hunt for it.

As water is unbearable, the Sulphur face is scaly and apt to be dirty-looking, careworn, and old.

It is often indicated in hectic fevers with circumscribed redness of the cheeks. Phos. has striking symptoms in the same condition. It is even stronger than Sulph.

We have every conceivable eruption upon the face—boils, impetigo, cracking and chapping of the lips, breaking of the skin under the alæ of the nose, under the lips, on the chin, rhagades, all associated with burning and cracking; skin burns upon application of *water*, foul taste in the mouth in the morning, after sleep, diarrhœa drives him out of bed as soon as he wakes, red tongue, red edges; red, dry, and burning in typhoid states. In this latter state it is very useful when you are called after an allopath has ptyalized his patient, and you will be glad to have known of Sulph.

Add to the characteristic symptoms already mentioned this: "A bad smell before the nose as of an old catarrh." Many remedies have fetid breath and bad smell from the mouth, but they differ in this way—this is entirely subjective—perceived by the patient himself, while others, like Merc., are simply abominable to every one about.

Nursing sore mouth of babies, thrush (Sulph. ac. It cures at least many cases) and we find it necessary to so prescribe, as it is impossible to find characteristics in such young children. ("Child screams violently upon downward motion." Borax. It is fear.) (Sensation "as of a worm in the bladder." Bell.)

There was a time when to hear such symptoms discussed seriously would have caused me to laugh, but since curing cases by these very symptoms I have felt that the laugh was against me.

Treasure up peculiar symptoms.

Tongue coated and wears off during the forenoon is more characteristic of *Merc.* It is also worse after sleep, showing the deeper derangements of the mucous membranes.

Sepia has the "burning of the pharynx."

Sulphur is similar to Lyc. in its sour eructations, burning all the way up into the throat and pharynx. Sulph. and Lyc. are sister remedies, and as Sulph. is followed by Calc., so is Calc. followed by Lyc.

The sore throat of Sulph. is like that of Lyc., going from right to left. Lach. produces a sore throat that moves from left to right. Sulph. ac. has a very characteristic sore throat, and is indicated in the lemon-colored exudations. Lemon-colored discharges are characteristic of this remedy. It has a stool looking like chopped lemon peel.

A singer at the Cave this summer was greatly troubled with tickling in his throat. Going to all the physicians in his neighborhood, all told him he must have the palate amputated! As a last resort he tried a despised homœopath—myself. I found a bright redness of palate and uvula, the latter looking like a little bag of water. A single dose of Kali-bich. cured the case.

The thirst of Sulph. is depraved, and is for alcohol or beer. This is in keeping with the state or condition in old, broken-down people. As they become old and feel a loss of strength they have cravings for strong drink.

Nux vom. is another remedy producing this condition, and is complementary to it. Podoph. and Nux v. antidote each other. Nux v. and Sulph. will do the same. Is it fear of harm to their patients that causes professed homœopaths to give the two together for piles?

A young lady patient of mine, having formed a habit of taking Chloral, was stopped by her parents. Slyly, without their knowledge, she began to take whisky and coffee. They became too poor for her to afford the whisky, so she had recourse to alcohol and coffee. She shut herself up, using twenty-five cents worth of alcohol every day!—a larger quantity than I ever knew to be used daily. Nux v. enabled that woman to stop drink immediately, and her nervousness also disappeared. Nux v., one dose per day for three months, then Sac. lac., cured her entirely. In this it is related to Sulph.

Marasmus, with great craving for food; "drinks much and eats little" when this violent thirst is on; there is but little appetite, and a large quantity of almost colorless urine is voided.

We have but one other strong remedy having "a ravenous desire for sweets, which make him sick"—Arg. n. These have both the desire and the *agg.*, but there is this distinction: The Sulph. patient will get up in the night to get sugar, which causes him to have sour stomach, heart-burn, vomiting, and a great amount of distress; Arg. n. will steal the sugar, and it brings on diarrhœa, or the mother will eat candy and her nursing baby will have a diarrhœa of "grass-green stools." You will think of Cham., of Acon., of Merc., but you must *give* Arg. n.

With a constipation, tenesmus, milk disagrees, causing sour taste and sour eructations, Colch. and Sep. are in good order. Puls. has sour eructations from milk and *fat* things. Each remedy has that about it which will enable you to individualize, has its own distinctive features and peculiarities, as each man is gifted with his own distinctive features and peculiarities.

